# Whole-Genome Sequencing and Annotation of a Clinical Isolate of *Mycobacterium tuberculosis* from Mumbai, India

**DOI:** 10.1128/genomeA.00154-14

**Published:** 2014-03-06

**Authors:** Ahmed Salim Ahmed Al Rashdi, Bhaskar L. Jadhav, Tejaswini Deshpande, Uday Deshpande

**Affiliations:** a Department of Life Sciences, Mumbai University, Mumbai, India; b Labindia-GPOD Research and Training Division, Thane, India

## Abstract

We report here the annotated genome sequence of a clinical isolate of *Mycobacterium tuberculosis* from Mumbai, India.

## GENOME ANNOUNCEMENT

The *Mycobacterium tuberculosis* complex (MTBC) lineages circulating in the Indian population are the Indo-Oceanic (IO), East African Indian (EAI), and Central Asian (CAS) (1–5). A study focusing on urban Mumbai and neighboring rural areas reported that, mainly, four types of *M. tuberculosis* strains, *viz*., Manu-1, CAS, Beijing, and East African Indian strains (EAI5), were found to be circulating in the population. Diversity of ancient and modern strains has been expected, with Mumbai being a world trend center (6). In the present research, clinical isolate *M. tuberculosis* MUM101 of the MTBC lineage circulating in the population of the Maharashtra state was subjected to whole-genome sequencing.

The paired-end sequencing was performed on an Illumina HiSeq platform. High-quality reads were mapped to the genome of reference strain *M. tuberculosis* H37Rv (accession no. NC_000962.2) using the CLC bio Genomics Workbench, as described earlier (7), generating a reference assembly with an average 45-read mapping coverage.

The NCBI Prokaryotic Genome Annotation Pipeline was used for the annotation of the reference assembly. The total numbers of genes and coding sequences (CDSs) identified are 4,026 and 3,902, respectively. Three types of rRNA, namely, 5S, 16S, and 23S, have been annotated. There are 45 tRNAs and 59 genes that exhibited frameshift mutations.

Further, using the software SpolPred, we determined the spoligotype of strain MUM101, which is 477777777413071, indicating that MUM101 belongs to the East African Indian lineage (EAI5).

### Nucleotide sequence accession number.

The annotated whole-genome sequence has been deposited in GenBank with the accession no. CP006578.
